# Associated factors of successful aging among community-dwelling older adults in a rural area of Indonesia: a cross-sectional study

**DOI:** 10.1590/1980-220X-REEUSP-2025-0577en

**Published:** 2026-08-24

**Authors:** Retno Indarwati, Joni Haryanto, Elida Ulfiana, Vira Amelia, Trihaningsih Puji Astuti, Deena Clare Thomas

**Affiliations:** 1 Universitas Airlangga, Faculty of Nursing, Gerontological Nursing Research Group, Surabaya, East Java, Indonesia.; 2 STIKes Bhakti Mulia, Professional Nursing Program, Kediri, East Java, Indonesia.; 3 Universiti Malaysia Sabah, Faculty of Medicine and Health Sciences, Department of Nursing, Kota Kinabalu, Sabah, Malaysia.

**Keywords:** Aged, Rural Population, Healthy Aging, Idoso, População Rural, Envelhecimento Saudável

## Abstract

**Objective:**

To identify the factors associated with successful aging among community-dwelling older adults in rural Indonesia.

**Method:**

This analytical cross-sectional study involved 104 older adults aged ≥60 years living in Lamongan Regency, Indonesia. Data were collected through structured face-to-face interviews assessing sociodemographic characteristics, health status, and five indicators of successful aging: physical function, cognitive function, psychological well-being, social engagement, and active engagement with life. Multiple linear regression analysis was performed to determine significant predictors.

**Results:**

Half of the respondents demonstrated moderate levels of successful aging. Working status (β = 0.383; 95% CI: 0.201–0.565; p < 0.001) and distance to health facilities (β = 0.588; 95% CI: 0.242–0.935; p = 0.001) were significant determinants. Older adults who were still working and those living 1–5 km from health facilities had higher successful aging scores.

**Conclusion:**

Successful aging among rural older adults is influenced by working status and proximity to healthcare facilities. Promoting productive activity and maintaining accessible, independent, and supportive health services are essential for aging well in rural communities.

## RESUMO

**Objetivo:**

Identificar os fatores associados ao envelhecimento bem-sucedidoentre idosos residentes na comunidade em área rural da Indonésia.

**Método:**

Estudo transversal analítico que envolveu 104 idosos com idade ≥60 anos residentes no Regency de Lamongan, Indonésia. Os dados foram coletados por meio de entrevistas presenciais estruturadas, avaliando características sociodemográficas, estado de saúde e cinco indicadores de envelhecimento bem-sucedidoentre: função física, função cognitiva, bem-estar psicológico, engajamento social e engajamento ativo com a vida. A análise de regressão linear múltipla foi realizada para determinar os preditores significativos.

**Resultados:**

Metade dos respondentes apresentou níveis moderados de envelhecimento bem-sucedidoentre. A situação laboral (β = 0,383; IC 95%: 0,201–0,565; p < 0,001) e a distância até os serviços de saúde (β = 0,588; IC 95%: 0,242–0,935; p = 0,001) foram determinantes significativos. Os idosos que ainda trabalhavam e aqueles que residiam a 1–5 km das unidades de saúde apresentaram escores mais elevados de envelhecimento bem-sucedidoentre.

**Conclusão:**

O envelhecimento bem-sucedidoentre entre idosos de áreas rurais é influenciado pela situação laboral e pela proximidade com os serviços de saúde. Promover a atividade produtiva e manter serviços de saúde acessíveis, independentes e de suporte são medidas essenciais para o envelhecimento bem-sucedido em comunidades rurais.

## INTRODUCTION

Population aging in Asia is increasing dramatically, with the number of adults aged 60 years and over projected to rise from 520 million to 1.2 billion by 2050^(1)^. A similar trend is occurring in Indonesia, where the population is also aging, with 19.2% of the population being older adults^(2)^. This phenomenon presents several challenges for governments and health systems, including the management of chronic diseases, long-term care, and social support^(3)^. In this context, the concept of successful aging has gained increasing relevance. As global demographic transitions continue to unfold, successful aging offers a multidimensional framework that highlights the importance of maintaining physical health, mental well-being, and active social engagement in later life, all of which contribute to optimal quality of life^(4,5)^.

Countries in low- and middle-income regions face significant challenges in promoting successful aging due to rapid demographic shifts, limited healthcare infrastructure, and socioeconomic disparities^(6,7)^. Older adults living in rural areas experience additional health burdens that are often intensified by geographical isolation and restricted access to healthcare services^(8)^. As a result, the health conditions affecting rural older adults are multifaceted, encompassing chronic diseases, mental health problems, functional impairments, and various social and environmental challenges^(9)^.

Promoting successful aging in Indonesia requires a comprehensive approach that addresses the multidimensional aspects of aging, supports healthy behaviors, and leverages social networks and community engagement^(10)^. Research on successful aging in Indonesia is not particularly rare, but it is still developing and expanding in scope, including prevalence and factors related to successful aging^(10)^; associated health behaviors and successful aging^(11)^; and perspectives on achieving successful aging^(12)^. However, studies specifically investigating the factors associated with successful aging among older adults living in rural areas remain limited. This gap is particularly important to address, given that the majority of older adults in Indonesia reside in rural settings, underscoring the need for research focused on the determinants of successful aging within this population.

## METHOD

### Study Design

Using an analytical cross-sectional survey model, this study was conducted with community-dwelling older adults aged 60 years and over living in Lamongan Regency, Indonesia, between August and September 2025. A total of 104 older adults were included in the study using a consecutive sampling method.

Analytical cross-sectional studies aim to measure exposures and outcomes at a single point in time to assess potential associations between variables. Although temporal causality cannot be determined, this design is appropriate for identifying correlational patterns and contributing factors of successful aging.

The inclusion criteria were: (1) age ≥60 years, (2) able to communicate verbally, (3) living in the community, and (4) willing to participate. Exclusion criteria were: (1) severe cognitive impairment, (2) bedridden, or (3) critical illness.

A prior power analysis was conducted using G*Power software to estimate the minimum sample size required for the regression analysis. Assuming a significance level of α = 0.05, a statistical power of 0.95 (1−β), and a medium effect size, the minimum required sample size was calculated before data collection. The final sample included 104 participants, meeting the required sample size and providing adequate statistical power to detect significant associations in the regression model.

### Data Collection

Data were collected using sociodemographic data, the successful aging assessment components (physical, cognitive, psychological, and social dimensions), and additional psychosocial variables related to family and spousal interactions. Data collection was conducted face-to-face in participants’ homes by trained research assistants following standardized procedures. Each interview session lasted approximately 40–60 minutes.

### Measurements

A structured questionnaire was developed by the researcher through a literature review. It consisted of 15 questions covering age, gender, education, marital status, working status, income, living arrangement, chronic illness history, medication use, health insurance status, and distance to the nearest healthcare facility. Items regarding spousal relationship satisfaction, daily interactions with spouse, spousal involvement in daily activities, and social engagement were also included.

Successful aging was assessed using five major components commonly used in gerontology research^(13)^. Health and Activities of Daily Living (ADL) were evaluated using the Short Physical Performance Battery (SPPB), which provides an objective measure of lower-extremity functional ability, including balance, gait speed, and chair-stand performance. Although SPPB does not directly assess basic ADLs, it is widely recognized as a valid indicator of physical functioning that strongly influences independence in daily living. Physical function was assessed using the Physical Activity Scale for the Elderly (PASE), which captures the frequency and duration of daily physical activities; higher scores indicate greater activity engagement. Cognitive function was measured using the 12-item General Health Questionnaire (GHQ-12), where higher scores indicate better cognitive and emotional stability. Psychological well-being was assessed using the Ryff Psychological Well-Being Scale (PWB), covering autonomy, environmental mastery, purpose in life, personal growth, positive relations, and self-acceptance. Active engagement with life was evaluated using the Being Actively Engaged in Life (BAEL) scale, with higher scores representing a stronger sense of meaningful involvement and future orientation. All five components were combined to generate a composite successful aging index, where higher values indicate a more optimal aging profile.

To construct the composite successful aging index, the scores from the five components (SPPB, PASE, GHQ-12, Ryff Psychological Well-Being Scale, and BAEL) were first standardized using z-score transformation to ensure comparability across different measurement scales. The standardized scores were then combined with equal weights to generate a single composite index, with higher scores indicating better successful aging. Equal weighting was applied to reflect the multidimensional nature of successful aging, in which each component is conceptually important and contributes equally to the overall construct. In addition, equal weighting was deemed appropriate because the instruments used to assess each component have different scoring ranges and measurement scales; therefore, z-score standardization was first applied to ensure comparability across components before aggregation.

The internal consistency of the composite index was assessed using Cronbach’s alpha to evaluate the coherence of the multidimensional construct. This approach is consistent with previous gerontological studies that conceptualize successful aging as a multidimensional phenomenon including physical functioning, psychological well-being, cognitive-emotional stability, and active engagement with life.

All instruments used in this study have been widely validated in previous research. The Indonesian versions of SPPB, PASE, GHQ-12, Ryff Psychological Well-Being Scale, and BAEL have been used in studies involving older adult populations.

### Statistical Analysis

Data were analyzed using the SPSS statistical package. Descriptive statistics (mean, standard deviation, frequency, percentage) were used to summarize participant characteristics. The Shapiro-Wilk test was used to assess normality. (1) Independent T-test and one-way ANOVA were performed for categorical predictors. (2) Pearson correlation analysis was used for continuous variables. (3) Variables with *p* < 0.05 from bivariate analysis were included in the multiple linear regression analysis (Enter model) to determine predictors of successful aging. Statistical significance was set at p < 0.05.

### Ethical Consideration

Prior to the study’s implementation, ethical approval was obtained from the Health Research Ethics Committee, Faculty of Nursing, Universitas Airlangga, with expedited review status. The study was approved under Ethical Approval Number: 3766-KEPK.

## RESULTS

The study involved 104 older adults with a mean age of 64.07 ± 4.6 years, with the majority being young-old (83.7%). The average length of marriage was 41.02 ± 6.6 years. The majority of respondents had completed primary education (71.2%), while only 3.8% had attained higher education. The proportion of male and female participants was equal (50% each). Most participants were still working (73.1%) and almost all respondents (98.1%) reported earnings below the regional minimum wage. Regarding living arrangements, 38.5% lived with their spouse and children, while 40.4% lived with extended family members such as in-laws or grandchildren. Almost half of the respondents (47.1%) reported no history of chronic diseases, 42.3% had fewer than two, and 10.6% had two or more. Regarding medication use, 59.6% were not taking any medication, and only 2.9% were taking 5 or more types. Most respondents reported being very satisfied with their relationship with their spouse (83.7%), with 71.2% spending time together all day. Spousal involvement in daily activities was generally high (68.3%). A majority were socially active in the community (80.8%) and maintained daily interactions with family members (95.2%). Furthermore, 85.6% were covered by the Indonesian National Health Insurance. Almost all respondents (94.2%) lived within 1–5 km of the nearest health facility, while only 5.8% lived less than 1 km away. Bivariate analysis showed that among all sociodemographic and health-related characteristics, only working status (p < 0.001) and distance to health facility (p = 0.001) were significantly associated with successful aging (Table 1). In contrast, variables such as age, gender, education, income, living arrangements, chronic disease history, medication use, spousal relationship satisfaction, and social involvement showed no significant association.

**Table 1:** Association between sociodemographic, health, and psychosocial characteristics with successful aging among older adults – Lamongan, Indonesia, 2025.

Variable	Total study population (N = 104)		p-value
	n	%	
Age (Mean ± SD)	64.07 ± 4.6		0.079^a^
Young-old (60–69 years)	87	83.6
Middle-old (70–79 years)	16	15.4
Old-old (80+ years)	1	1.0
Length of marriage (Mean ± SD)	41.02 ± 6.6		0.423^a^
Education			
No formal education	6	5.8	0.139^b^
Primary school	74	71.2
Secondary school	20	19.2
Higher education	4	3.8
Gender			
Female	52	50.0	0.142^c^
Male	52	50.0
Working status			
Working	76	73.1	**<0.001^c^**
Not working	28	26.9
Income			
Below minimum wage	102	98.1	0.473^c^
Above minimum wage	2	1.9
Living arrangement			
Only with spouse	20	19.2	0.174^b^
Spouse and children	40	38.5
Spouse, children, in-laws, and grandchildren	2	1.9
Others	42	40.4
History of chronic diseases			
None	49	47.1	0.137^b^
< 2 diseases	44	42.3
≥ 2 diseases	11	10.6
Medication use			
Not taking any medication	62	59.6	0.205^b^
< 5 types	39	37.5
≥ 5 types	3	2.9
Satisfaction with relationship with spouse			
Satisfied	17	16.3	0.213^c^
Very satisfied	87	83.7
Frequency of daily interaction with spouse			
Rarely together	2	1.9	0.878^b^
A few hours	28	26.9
Together all day	74	71.2
Spousal involvement in daily activities			
Low	9	8.6	0.116^b^
Moderate	24	23.1
High	71	68.3
Social involvement in community			
No	20	19.2	0.661^c^
Yes	84	80.8
Social involvement with family			
Monthly	1	1.0	0.223^b^
Weekly	4	3.8
Daily	99	95.2
Health insurance			
None	15	14.4	0.713^c^
Indonesian national health insurance	89	85.6
Distance to health facility			
< 1 KM	6	5.8	**0.001^c^**
1–5 KM	98	94.2	

*p-value* < 0.05. ^a^Pearson test

^b^One-way ANOVA test;

^c^Independent T-Test;

KM: kilometer.

Based on Table 2, the mean physical function score was 167.1 ± 108.6. For ADL, 51.9% had mild limitations and 35.6% had minimal limitations. Cognitive function was moderate in 72.1% and high in 27.9%. Most participants were highly engaged in life (77.9%), with 21.1% moderately engaged. Psychological well-adapted was moderate in 44.2% and high in 33.7%. Overall, 50.0% were classified as having moderate Successful Aging, while 25.0% each were low or high.

**Table 2:** Successful aging and its indicators among older adults – Lamongan, Indonesia, 2025.

Indicator	N = 104	%
Physical function (Mean ± SD) PASE	167.1 ± 108.6	
Activity Daily Living	
Severe limitation	3	2.9
Moderate limitation	10	9.6
Mild limitation	54	51.9
Minimal limitation	37	35.6
Cognitive function	
Moderate	75	72.1
High	29	27.9
Being Actively Engaged in Life	
0	1	1.0
1	22	21.1
2	81	77.9
Psychological well adapted	
Low	23	22.1
Moderate	46	44.2
High	35	33.7
Overall successful Aging	
Low	26	25.0
Moderate	52	50.0
High	26	25.0

Table 3 shows the factors associated with successful aging among older adults. The analysis revealed that working status and distance to health facilities were significant determinants of successful aging. Older adults who were still working had higher successful aging scores compared to those who were not working (β = 0.383; 95% CI: 0.201–0.565; p < 0.001). Similarly, respondents who lived 1–5 km from health facilities had higher successful aging scores compared to those living closer than 1 km (β = 0.588; 95% CI: 0.242–0.935; p = 0.001).

**Table 3:** Associated factors of successful aging among older adults – Lamongan, Indonesia, 2025.

Variable	Unstandardized Coefficients		Standardized Coefficients	CI-95%	*p-value*
	*B*	Std. Error	*β*
Constant	–0.834	0.180		–1.192 – –0.447	<0.001
Working Status				
Not working (ref.)	1
Working	0.383	0.092	0.365	0.201 – 0.565	<0.001
Distance to Health Facility				
<1 KM (ref.)	1
1–5 KM	0.588	0.175	0.295	0.242 – 0.935	0.001
Adjusted R^2^	0.212				

*p-value* < 0.05. B: unstandardized regression coefficient; β: standardized regression coefficient; CI: confidence interval; Adj. R^2^: adjusted coefficient of determination; Ref.: reference category; KM: kilometer.

## DISCUSSION

The demographic characteristics of the participants in this study indicate that most older adults were in the young-old category, had long marital durations, lived in multigenerational households, and maintained strong interactions with spouses and extended family members. These demographic patterns are commonly observed in rural communities across several countries, including China, where older adults living in rural areas tend to have stronger family cohesion, rely more on intergenerational support, and sustain traditional family structures compared to their urban counterparts^(14,15)^. Despite low educational attainment and income levels, the majority of participants reported relatively good health status, minimal medication use, and high involvement in community and family activities. Similar demographic patterns have been documented in studies from rural Indonesia and other ASEAN countries, such as Malaysia, Thailand, and Vietnam, where strong intergenerational bonds and collectivist cultural norms help sustain emotional well-being and functional independence among older adults, even in the context of socioeconomic disadvantage^(16,17)^. These findings suggest that rural family structures provide a significant buffering effect that supports successful aging. In our view, this pattern reflects the resilience and embedded support systems within rural communities, which may compensate for limited economic resources and formal education.

Regarding the multidimensional components of successful aging, older adults in this study demonstrated favorable scores across physical, cognitive, and psychosocial dimensions. Most participants exhibited mild-to-minimal ADL limitations, moderate-to-high cognitive functioning, strong life engagement, and good psychological adaptation. These results align with evidence from rural studies in China^(18,19)^ and Vietnam^(20)^, which show that daily physical activity, communal participation, and close social networks contribute to sustaining functional and cognitive health in older adults. In the specific rural context of Lamongan, frequent daily interactions with family members and active involvement in community routines likely serve as natural facilitators of psychological resilience and cognitive stimulation. This suggests that rural environmental and cultural conditions may inherently support the maintenance of successful aging indicators.

The findings of this study show that older adults have moderate successful aging. This result aligns with previous studies reporting a moderate level of active aging^(21)^. Factors such as working status and distance to health facilities are associated with successful aging among older adults. Older adults who are working have higher levels of successful aging than those who are not working. This finding is in line with Choi et al.^(22)^, who found that older workers are significantly associated with their cognitive functioning. Furthermore, employment offers older adults’ meaningful opportunities for social engagement and interpersonal support, both of which play an essential role in promoting successful aging^(23)^. Remaining employed also facilitates broader social participation, which in turn supports better mental health^(24)^.

Interestingly, distance to health facilities also emerged as a significant factor, with those living 1–5 km away reporting higher levels of successful aging than those living within 1 km. This finding should be interpreted cautiously, as it remains exploratory and does not imply a causal relationship. Given the cross-sectional design, it is not possible to determine whether distance itself influences successful aging or reflects underlying differences in health status, mobility, or environmental context. The result differs from the common assumption that closer proximity to healthcare services universally benefits older adults^(25,26,27)^. One possible explanation, which should be interpreted with caution, is that older adults living slightly farther away may maintain higher mobility, independence, and community engagement as part of their routine in accessing services, which could indirectly support their physical and psychological functioning. However, this interpretation is speculative and was not directly assessed in this study, suggesting that distance to health facilities may reflect broader contextual and health-related conditions rather than access alone.

This study has limitations that should be considered when interpreting the findings. The study was conducted in a single rural district, which may limit the generalizability of the findings to urban or other rural populations with different cultural and socioeconomic characteristics. Participants were recruited using a non-probabilistic consecutive sampling method, which may introduce selection bias and limit the sample’s representativeness of the broader population. Some categories included a relatively small number of participants, such as respondents aged 80 years or older, those living less than 1 km from a health facility, and those with an income above the minimum wage. The imbalance in these categories may affect the stability of the statistical estimates and should therefore be interpreted with caution. The adjusted R^2^ value (0.212) further indicates that the model explains a relatively limited proportion of the variance in successful aging, suggesting that other unmeasured factors may also play an important role. Moreover, the sample showed limited variability in key characteristics, particularly income and distance to health facilities, with most participants clustered within similar categories. This homogeneity may reduce the analysis’s discriminatory power and affect the stability and generalizability of the regression estimates.

## CONCLUSION

This study highlights that most older adults in rural areas demonstrate moderate levels of successful aging. Among the sociodemographic and health-related factors examined, working status and distance to health facilities were significantly associated with successful aging. Older adults who remained employed and those living 1–5 km from health facilities exhibited higher successful aging scores. These findings emphasize the importance of encouraging productive engagement in later life and of maintaining accessible, yet independence-promoting, health services to support aging well in rural contexts. However, due to the cross-sectional design of this study, causal relationships cannot be established. In addition, we have also noted that the ratio of participants to predictors may limit model stability, and results should therefore be interpreted with caution. Future research should explore longitudinal and qualitative approaches to understand better the mechanisms underlying these associations.

## Data Availability

The entire dataset supporting the results of this study was published in the article itself. Additional data are available from the corresponding author upon reasonable request and subject to confidentiality considerations.
