# Supplementary material for: Associated factors of successful aging among community-dwelling older adults in a rural area of Indonesia: a cross-sectional study
Source: Rev Esc Enferm USP. 2026 Aug 24;60:e20250577. doi: 10.1590/1980-220X-REEUSP-2025-0577en (PMC13502945; doi:10.1590/1980-220X-REEUSP-2025-0577en)
Supplement: Translation

# Translation: Factores asociados al envejecimiento exitoso entre adultos mayores residentes en la comunidad en un área rural de Indonesia: estudio transversal

**DOI:** 10.1590/1980-220X-REEUSP-2025-0577es

**Authors:** Retno Indarwati, Joni Haryanto, Elida Ulfiana, Vira Amelia, Trihaningsih Puji Astuti, Deena Clare Thomas

**Affiliations:** 1 Universitas Airlangga, Faculty of Nursing, Gerontological Nursing Research Group, Surabaya, East Java, Indonesia.; 2 STIKes Bhakti Mulia, Professional Nursing Program, Kediri, East Java, Indonesia.; 3 Universiti Malaysia Sabah, Faculty of Medicine and Health Sciences, Department of Nursing, Kota Kinabalu, Sabah, Malaysia.

**Keywords:** Anciano, Población Rural, Envejecimiento Saludable

## Abstract

**Objetivo:**

Identificar los factores asociados al envejecimiento exitoso entre adultos mayores residentes en la comunidad en un área rural de Indonesia.

**Método:**

Estudio transversal analítico que involucró a 104 adultos mayores con edad ≥60 años residentes en el Regency de Lamongan, Indonesia. Los datos fueron recolectados mediante entrevistas presenciales estructuradas que evaluaron características sociodemográficas, estado de salud y cinco indicadores de envejecimiento exitoso: función física, función cognitiva, bienestar psicológico, participación social y participación activa en la vida. Se realizó un análisis de regresión lineal múltiple para determinar los predictores significativos.

**Resultados:**

La mitad de los encuestados presentó niveles moderados de envejecimiento exitoso. La situación laboral (β = 0,383; IC 95%: 0,201–0,565; p < 0,001) y la distancia a los servicios de salud (β = 0,588; IC 95%: 0,242–0,935; p = 0,001) fueron determinantes significativos. Los adultos mayores que aún trabajaban y aquellos que vivían a 1–5 km de las instalaciones de salud obtuvieron puntajes más elevados de envejecimiento exitoso.

**Conclusión:**

El envejecimiento exitoso entre los adultos mayores de áreas rurales está influenciado por la situación laboral y la proximidad a los servicios de salud. Promover la actividad productiva y mantener servicios de salud accesibles, independientes y de apoyo son medidas esenciales para envejecer bien en comunidades rurales.

## INTRODUCCIÓN

El envejecimiento de la población en Asia está aumentando drásticamente, y se prevé que el número de adultos de 60 años o más aumente de 520 millones a 1200 millones para 2050^(1)^. Se está produciendo una tendencia similar en Indonesia, donde la población también está envejeciendo, con un 19,2% de la población compuesta por adultos mayores^(2)^. Este fenómeno plantea varios desafíos para los gobiernos y los sistemas de atención de salud, entre ellos la gestión de las enfermedades crónicas, los cuidados a largo plazo y el apoyo social^(3)^. En este contexto, el concepto de envejecimiento exitoso ha cobrado cada vez más relevancia. A medida que continúan las transiciones demográficas a nivel mundial, el envejecimiento exitoso ofrece un marco multidimensional que destaca la importancia de mantener la salud física, el bienestar mental y la participación social activa en la tercera edad, factores que contribuyen a una calidad de vida óptima^(4,5)^.

Los países de las regiones de ingresos bajos y medios enfrentan desafíos significativos para promover el envejecimiento exitoso debido a los rápidos cambios demográficos, la infraestructura sanitaria limitada y las disparidades socioeconómicas^(6,7)^. Las personas mayores que viven en zonas rurales experimentan cargas de salud adicionales que a menudo se ven intensificadas por el aislamiento geográfico y el acceso restringido a los servicios de salud^(8)^. Como resultado, las condiciones de salud que afectan a las personas mayores rurales son multifacéticas y abarcan enfermedades crónicas, problemas de salud mental, discapacidades funcionales y diversos desafíos sociales y ambientales^(9)^.

Promover el envejecimiento exitoso en Indonesia requiere un enfoque integral que aborde los aspectos multidimensionales del envejecimiento, apoye los comportamientos saludables y aproveche las redes sociales y la participación comunitaria^(10)^. La investigación sobre el envejecimiento satisfactorio en Indonesia no es particularmente escasa, pero aún se encuentra en desarrollo y ampliando su alcance, incluyendo la prevalencia y los factores relacionados con el envejecimiento satisfactorio; los comportamientos de salud asociados y el envejecimiento satisfactorio^(11)^; y las perspectivas sobre cómo lograr un envejecimiento satisfactorio. Sin embargo, los estudios que investigan específicamente los factores asociados con el envejecimiento satisfactorio^(12)^ entre las personas mayores que viven en zonas rurales siguen siendo limitados. Es particularmente importante abordar esta brecha, dado que la mayoría de las personas mayores en Indonesia residen en entornos rurales, lo que subraya la necesidad de investigaciones centradas en los determinantes del envejecimiento satisfactorio dentro de esta población.

## MÉTODO

### Diseño del Estudio

Utilizando un modelo de encuesta transversal analítica, este estudio se llevó a cabo con personas mayores de 60 años o más que vivían en la comunidad en la regencia de Lamongan, Indonesia, entre agosto y septiembre de 2025. Se incluyó en el estudio a un total de 104 adultos mayores mediante un método de muestreo consecutivo.

Los estudios analíticos transversales tienen como objetivo medir las exposiciones y los resultados en un momento específico para evaluar posibles asociaciones entre variables. Aunque no se puede determinar la causalidad temporal, este diseño es adecuado para identificar patrones correlacionales y factores que contribuyen al envejecimiento exitoso.

Los criterios de inclusión fueron: (1) edad ≥60 años, (2) capacidad para comunicarse verbalmente, (3) vivir en la comunidad y (4) estar dispuesto a participar. Los criterios de exclusión fueron: (1) deterioro cognitivo grave, (2) estar postrado en cama o (3) padecer una enfermedad crítica.

Se realizó un análisis de potencia previo utilizando el software G*Power para estimar el tamaño mínimo de la muestra requerido para el análisis de regresión. Suponiendo un nivel de significación de α = 0,05, una potencia estadística de 0,95 (1−β) y un tamaño del efecto medio, se calculó el tamaño mínimo de la muestra requerido antes de la recolección de datos. La muestra final incluyó a 104 participantes, lo que cumplió con el tamaño de muestra requerido y proporcionó la potencia estadística adecuada para detectar asociaciones significativas en el modelo de regresión.

### Recolección de Datos

Los datos se recolectaron utilizando datos sociodemográficos, los componentes de la evaluación del envejecimiento satisfactorio (dimensiones física, cognitiva, psicológica y social) y variables psicosociales adicionales relacionadas con las interacciones familiares y conyugales. La recolección de datos se llevó a cabo en persona en los hogares de los participantes por parte de asistentes de investigación capacitados, siguiendo procedimientos estandarizados. Cada sesión de entrevista duró aproximadamente entre 40 y 60 minutos.

### Mediciones

El investigador elaboró un cuestionario estructurado a partir de una revisión bibliográfica. Consistía en 15 preguntas que abarcaban la edad, el género, la educación, el estado civil, la situación laboral, los ingresos, la situación de vivienda, los antecedentes de enfermedades crónicas, el uso de medicamentos, la situación del seguro médico y la distancia al centro de salud más cercano. También se incluyeron ítems relativos a la satisfacción con la relación conyugal, las interacciones diarias con el cónyuge, la participación del cónyuge en las actividades diarias y la participación social.

El envejecimiento satisfactorio se evaluó utilizando cinco componentes principales comúnmente utilizados en la investigación gerontológica^(13)^. La salud y las actividades de la vida diaria (AVD) se evaluaron mediante la Batería Corta de Rendimiento Físico (Short Physical Performance Battery - SPPB), que proporciona una medida objetiva de la capacidad funcional de las extremidades inferiores, incluyendo el equilibrio, la velocidad de la marcha y el rendimiento al levantarse de una silla. Aunque la SPPB no evalúa directamente las AVD básicas, es ampliamente reconocida como un indicador válido del funcionamiento físico que influye fuertemente en la independencia en la vida diaria.

La función física se evaluó mediante la Escala de Actividad Física para Adultos Mayores (Physical Activity Scale for the Elderly - PASE), que capta la frecuencia y la duración de las actividades físicas diarias; las puntuaciones más altas indican una mayor participación en actividades.

La función cognitiva se midió mediante el Cuestionario de Salud General de 12 ítems (General Health Questionnaire - GHQ-12), en el que las puntuaciones más altas indican una mejor estabilidad cognitiva y emocional. El bienestar psicológico se evaluó mediante la Escala de Bienestar Psicológico de Ryff (Psychological Well-Being Scale - PWB), que abarca la autonomía, el dominio del entorno, el propósito en la vida, el crecimiento personal, las relaciones positivas y la autoaceptación.

La participación activa en la vida se evaluó mediante la escala de Participación Activa en la Vida (Being Actively Engaged in Life - BAEL), en la que las puntuaciones más altas representan un mayor sentido de participación significativa y orientación hacia el futuro. Los cinco componentes se combinaron para generar un índice compuesto de envejecimiento exitoso, en el que los valores más altos indican un perfil de envejecimiento más óptimo.

Para construir el índice compuesto de envejecimiento exitoso, las puntuaciones de los cinco componentes (SPPB, PASE, GHQ-12, Escala de Bienestar Psicológico de Ryff y BAEL) se estandarizaron primero mediante la transformación de la puntuación z para garantizar la comparabilidad entre las diferentes escalas de medición. A continuación, las puntuaciones estandarizadas se combinaron con ponderaciones iguales para generar un único índice compuesto, en el que las puntuaciones más altas indican un envejecimiento más exitoso. Se aplicó una ponderación igual para reflejar la naturaleza multidimensional del envejecimiento exitoso, en la que cada componente es conceptualmente importante y contribuye por igual al constructo general.

Además, se consideró apropiada la ponderación equitativa porque los instrumentos utilizados para evaluar cada componente tienen diferentes rangos de puntuación y escalas de medición; por lo tanto, se aplicó primero la estandarización mediante la puntuación z para garantizar la comparabilidad entre los componentes antes de la agregación.

La consistencia interna del índice compuesto se evaluó utilizando el alfa de Cronbach para evaluar la coherencia del constructo multidimensional. Este enfoque es coherente con estudios gerontológicos previos que conceptualizan el envejecimiento exitoso como un fenómeno multidimensional que incluye el funcionamiento físico, el bienestar psicológico, la estabilidad cognitivo-emocional y la participación activa en la vida.

Todos los instrumentos utilizados en este estudio han sido ampliamente validados en investigaciones previas. Las versiones indonesias del SPPB, el PASE, el GHQ-12, la Escala de Bienestar Psicológico de Ryff y el BAEL se han utilizado en estudios con poblaciones de adultos mayores.

### Análisis Estadístico

Los datos se analizaron utilizando el paquete estadístico SPSS. Se utilizaron estadísticas descriptivas (media, desviación estándar, frecuencia, porcentaje) para resumir las características de los participantes. Se utilizó la prueba de Shapiro-Wilk para evaluar la normalidad. (1) Se realizaron pruebas T independientes y ANOVA unidireccionales para los predictores categóricos. (2) Se utilizó el análisis de correlación de Pearson para las variables continuas. (3) Las variables con *p* < 0,05 del análisis bivariado se incluyeron en el análisis de regresión lineal múltiple (modelo Enter) para determinar los predictores del envejecimiento exitoso. La significación estadística se fijó en p < 0,05.

### Consideraciones Éticas

Antes de la implementación del estudio, se obtuvo la aprobación ética del Comité de Ética en Investigación en Salud de la Facultad de Enfermería de la Universidad Airlangga, con estatus de revisión acelerada. El estudio fue aprobado con el número de autorización ética: 3766-KEPK.

## Resultados

En el estudio participaron 104 adultos mayores con una edad media de 64,07 ± 4,6 años, siendo la mayoría adultos mayores jóvenes (83,7%). La duración promedio del matrimonio fue de 41,02 ± 6,6 años. La mayoría de los encuestados había completado la educación primaria (71,2%), mientras que solo el 3,8% había alcanzado la educación superior. La proporción de participantes hombres y mujeres fue igual (50% cada uno). La mayoría de los participantes aún trabajaba (73,1%) y casi todos los encuestados (98,1%) informaron ingresos por debajo del salario mínimo regional. En cuanto a la situación de convivencia, el 38,5% vivía con su cónyuge e hijos, mientras que el 40,4% vivía con miembros de la familia extensa, como suegros o nietos. Casi la mitad de los encuestados (47,1%) declaró no tener antecedentes de enfermedades crónicas, el 42,3% tenía menos de dos y el 10,6% tenía dos o más. En cuanto al uso de medicamentos, el 59,6% no tomaba ningún medicamento, y solo el 2,9% tomaba 5 o más tipos. La mayoría de los encuestados declaró estar muy satisfechos con su relación con su cónyuge (83,7%), y el 71,2% pasaba tiempo juntos todo el día. La participación del cónyuge en las actividades diarias fue, en general, alta (68,3%). La mayoría era socialmente activa en la comunidad (80,8%) y mantenía interacciones diarias con los miembros de la familia (95,2%). Además, el 85,6% estaba cubierto por el Seguro Nacional de Salud de Indonesia. Casi todos los encuestados (94,2%) vivían a una distancia de entre 1 y 5 km del centro de salud más cercano, mientras que solo el 5,8% vivía a menos de 1 km. El análisis bivariado mostró que, entre todas las características sociodemográficas y relacionadas con la salud, solo la situación laboral (p < 0,001) y la distancia al centro de salud (p = 0,001) se asociaron significativamente con el envejecimiento satisfactorio (Tabla 1). Por el contrario, variables como la edad, el género, la educación, los ingresos, las condiciones de vivienda, los antecedentes de enfermedades crónicas, el uso de medicamentos, la satisfacción con la relación conyugal y la participación social no mostraron una asociación significativa.

**Tabla 1:** Asociación entre las características sociodemográficas, de salud y psicosociales y el envejecimiento satisfactorio entre las personas mayores – Lamongan, Indonesia, 2025.

Variable	Población total del estudio (N = 104)		Valor p
	n	%
Edad (media ± DE)	64,07 ± 4,6		0,079^a^
Jóvenes mayores (60-69 años)	87	83,6
Mayores de mediana edad (70-79 años)	16	15,4
Tercera edad (80+ años)	1	1,0
Duración del matrimonio (media ± DE)	41,02 ± 6,6		0,423^a^
Nivel educativo			
Sin educación formal	6	5,8	0,139^b^
Educación primari^a^	74	71,2
Educación secundari^a^	20	19,2
Educación superior	4	3,8
Género			
Mujeres	52	50,0	0,142^c^
Hombre	52	50,0
Situación laboral			
Activo	76	73,1	**<0,001** ^c^
Sin empleo	28	26,9
Ingresos			
Por debajo del salario mínimo	102	98,1	0,473^c^
Por encima del salario mínimo	2	1,9
Condiciones de viviend^a^			
Solo con el cónyuge	20	19,2	0,174^b^
Cónyuge e hijos	40	38,5
Cónyuge, hijos, suegros y nietos	2	1,9
Otros	42	40,4
Antecedentes de enfermedades crónicas			
Ningun^a^	49	47,1	0,137^b^
< 2 enfermedades	44	42,3
≥ 2 enfermedades	11	10,6
Uso de medicamentos			
No toma ningún medicamento	62	59,6	0,205^b^
< 5 tipos	39	37,5
≥ 5 tipos	3	2,9
Satisfacción con la relación con el cónyuge			
Satisfecho	17	16,3	0,213^c^
Muy satisfecho	87	83,7
Frecuencia de la interacción diaria con el cónyuge			
Rara vez juntos	2	1,9	0,878^b^
Unas pocas horas	28	26,9
Juntos todo el dí^a^	74	71,2
Participación del cónyuge en las actividades diarias			
Baj^a^	9	8,6	0,116^b^
Moderad^a^	24	23,1
Alto	71	68,3
Participación social en la comunidad			
No	20	19,2	0,661^c^
Sí	84	80,8
Participación social con la famili^a^			
Mensual	1	1,0	0,223^b^
Semanal	4	3,8
Diario	99	95,2
Seguro médico			
Ninguno	15	14,4	0,713^c^
Seguro nacional de salud de Indonesi^a^	89	85,6
Distancia al centro de salud			
< 1 km	6	5,8	**0,001** ^c^
1–5 km	98	94,2	

*valor p* < 0,05. ^a^Prueba de Pearson; ^b^Prueba ANOVA unidireccional; ^c^Prueba T independiente; KM: kilómetro.

Según la Tabla 2, la puntuación media de la función física fue de 167,1 ± 108,6. En cuanto a las AVD, el 51,9% presentaba limitaciones leves y el 35,6% presentaba limitaciones mínimas. La función cognitiva fue moderada en el 72,1% y alta en el 27,9%. La mayoría de los participantes mostraban un alto nivel de implicación en la vida (77,9%), mientras que el 21,1% presentaba un nivel de implicación moderado. La adaptación psicológica fue moderada en el 44,2% y alta en el 33,7%. En general, el 50,0% fue clasificado como con un envejecimiento satisfactorio moderado, mientras que el 25,0% correspondía a cada uno de los niveles bajo o alto.

**Tabla 2:** Envejecimiento satisfactorio y sus indicadores entre las personas mayores – Lamongan, Indonesia, 2025.

Indicador	N = 104	%
Función física (media ± DE) PASE	167,1 ± 108,6	
Actividades de la vida diaria			
Limitación grave	3	2,9
Limitación moderada	10	9,6
Limitación leve	54	51,9
Limitación mínima	37	35,6
Función cognitiva			
Moderada	75	72,1
Alta	29	27,9
Participar activamente en la vida			
0	1	1,0
1	22	21,1
2	81	77,9
Bien adaptado psicológicamente			
Bajo	23	22,1
Moderado	46	44,2
Alto	35	33,7
Envejecimiento satisfactorio en general			
Bajo	26	25,0
Moderado	52	50,0
Alto	26	25,0

La Tabla 3 muestra los factores asociados con el envejecimiento exitoso entre las personas mayores. El análisis reveló que la situación laboral y la distancia a los servicios de salud fueron determinantes significativos del envejecimiento exitoso. Las personas mayores que aún trabajaban obtuvieron puntuaciones más altas de envejecimiento exitoso en comparación con aquellas que no trabajaban (β = 0,383; IC del 95%: 0,201–0,565; p < 0,001). Del mismo modo, los participantes que vivían a una distancia de 1–5 km de los servicios de salud obtuvieron puntuaciones más altas de envejecimiento exitoso en comparación con quienes vivían a menos de 1 km (β = 0,588; IC del 95%: 0,242–0,935; p = 0,001).

**Tabla 3:** Factores asociados al envejecimiento satisfactorio entre las personas mayores – Lamongan, Indonesia, 2025.

Variable	Coeficientes no estandarizados		Coeficientes estandarizados	IC del 95%	*Valor p*
	*B*	Error estándar	*β*
Constante	–0,834	0,180		–1,192 – –0,447	<0,001
Estado de funcionamiento			
Sin empleo (ref.)	1
Trabajando	0,383	0,092	0,365	0,201–0,565	<0,001
Distancia al centro sanitario			
<1 km (ref.)	1
1–5 km	0,588	0,175	0,295	0,242 – 0,935	0,001
R^2^ajustado	0,212				

valor p < 0,05. B: coeficiente de regresión no estandarizado; β: coeficiente de regresión estandarizado; IC: intervalo de confianza; R^2^ ajustado: coeficiente de determinación ajustado; Ref.: categoría de referencia; KM: kilómetro.

## DISCUSIÓN

Las características demográficas de los participantes en este estudio indican que la mayoría de los adultos mayores se encontraban en la categoría de adultos mayores jóvenes, tenían matrimonios de larga duración, vivían en hogares multigeneracionales y mantenían fuertes interacciones con sus cónyuges y familiares de la familia extensa. Estos patrones demográficos se observan comúnmente en comunidades rurales de varios países, incluida China, donde los adultos mayores que viven en zonas rurales tienden a tener una mayor cohesión familiar, dependen más del apoyo intergeneracional y mantienen estructuras familiares tradicionales en comparación con sus contrapartes urbanas^(14,15)^. A pesar de los bajos niveles de escolaridad e ingresos, la mayoría de los participantes informaron de un estado de salud relativamente bueno, un uso mínimo de medicamentos y una alta participación en actividades comunitarias y familiares. Se han documentado patrones demográficos similares en estudios realizados en zonas rurales de Indonesia y otros países de la ASEAN, como Malasia, Tailandia y Vietnam, donde los fuertes lazos intergeneracionales y las normas culturales colectivistas ayudan a mantener el bienestar emocional y la independencia funcional entre las personas mayores, incluso en un contexto de desventaja socioeconómica^(16,17)^.

Estos hallazgos sugieren que las estructuras familiares rurales proporcionan un efecto amortiguador significativo que favorece el envejecimiento satisfactorio. En nuestra opinión, este patrón refleja la resiliencia y los sistemas de apoyo arraigados en las comunidades rurales, que pueden compensar los recursos económicos limitados y la educación formal.

En cuanto a los componentes multidimensionales del envejecimiento satisfactorio, las personas mayores de este estudio obtuvieron puntuaciones favorables en las dimensiones física, cognitiva y psicosocial. La mayoría de los participantes mostraron limitaciones leves a mínimas en las actividades de la vida diaria (AVD), un funcionamiento cognitivo de moderado a alto, una fuerte participación en la vida y una buena adaptación psicológica. Estos resultados concuerdan con la evidencia de estudios rurales en China^(18,19)^ y Vietnam^(20)^, que muestran que la actividad física diaria, la participación comunitaria y las redes sociales cercanas contribuyen a mantener la salud funcional y cognitiva en las personas mayores. En el contexto rural específico de Lamongan, las interacciones diarias frecuentes con los miembros de la familia y la participación activa en las rutinas de la comunidad probablemente sirvan como facilitadores naturales de la resiliencia psicológica y la estimulación cognitiva. Esto sugiere que las condiciones ambientales y culturales rurales pueden apoyar de manera inherente el mantenimiento de los indicadores de envejecimiento exitoso.

Los hallazgos de este estudio muestran que las personas mayores presentan un envejecimiento exitoso moderado^(21)^. Este resultado concuerda con estudios previos que reportan un nivel moderado de envejecimiento activo. Factores como la situación laboral y la distancia a los centros de salud se asocian con el envejecimiento satisfactorio entre las personas mayores^(21)^. Las personas mayores que trabajan presentan niveles más altos de envejecimiento satisfactorio que aquellas que no lo hacen. Este hallazgo concuerda con el de Choi et al.^(22)^, quienes encontraron que los trabajadores mayores están significativamente asociados con su funcionamiento cognitivo. Además, el empleo ofrece a las personas mayores oportunidades significativas de participación social y apoyo interpersonal, los cuales desempeñan un papel esencial en la promoción del envejecimiento satisfactorio^(23)^. Mantenerse en el empleo también facilita una mayor participación social, lo que a su vez favorece una mejor salud mental^(24)^.

Curiosamente, la distancia a los centros de salud también se reveló como un factor significativo, ya que quienes viven a una distancia de 1 a 5 km reportaron niveles más altos de envejecimiento satisfactorio que quienes viven a menos de 1 km. Este hallazgo debe interpretarse con cautela, ya que sigue siendo exploratorio y no implica una relación causal. Dado el diseño transversal, no es posible determinar si la distancia en sí misma influye en el envejecimiento satisfactorio o si refleja diferencias subyacentes en el estado de salud, la movilidad o el contexto ambiental. El resultado difiere de la suposición común de que una mayor proximidad a los servicios de salud beneficia universalmente a las personas mayores^(25,26,27)^. Una posible explicación, que debe interpretarse con cautela, es que las personas mayores que viven un poco más lejos pueden mantener una mayor movilidad, independencia y participación en la comunidad como parte de su rutina al acceder a los servicios, lo que podría apoyar indirectamente su funcionamiento físico y psicológico. Sin embargo, esta interpretación es especulativa y no se evaluó directamente en este estudio, lo que sugiere que la distancia a los centros de salud puede reflejar condiciones contextuales y relacionadas con la salud más amplias, en lugar del acceso por sí solo.

Este estudio tiene limitaciones que deben tenerse en cuenta al interpretar los hallazgos. El estudio se llevó a cabo en un solo distrito rural, lo que puede limitar la generalización de los hallazgos a poblaciones urbanas u otras poblaciones rurales con características culturales y socioeconómicas diferentes. Los participantes fueron reclutados mediante un método de muestreo consecutivo no probabilístico, lo que puede introducir un sesgo de selección y limitar la representatividad de la muestra de la población general. Algunas categorías incluyeron un número relativamente pequeño de participantes, como los encuestados de 80 años o más, los que vivían a menos de 1 km de un centro de salud y los que tenían ingresos superiores al salario mínimo. El desequilibrio en estas categorías puede afectar la estabilidad de las estimaciones estadísticas y, por lo tanto, debe interpretarse con cautela. El valor R^2^ ajustado (0,212) indica además que el modelo explica una proporción relativamente limitada de la varianza en el envejecimiento satisfactorio, lo que sugiere que otros factores no medidos también pueden desempeñar un papel importante. Además, la muestra mostró una variabilidad limitada en características clave, particularmente los ingresos y la distancia a los centros de salud, con la mayoría de los participantes agrupados en categorías similares. Esta homogeneidad puede reducir el poder discriminatorio del análisis y afectar la estabilidad y la generalizabilidad de las estimaciones de regresión.

## CONCLUSIÓN

Este estudio destaca que la mayoría de los adultos mayores en zonas rurales muestran niveles moderados de envejecimiento exitoso. Entre los factores sociodemográficos y relacionados con la salud examinados, la situación laboral y la distancia a los centros de salud se asociaron significativamente con el envejecimiento exitoso. Los adultos mayores que permanecieron empleados y aquellos que vivían a una distancia de 1 a 5 km de los centros de salud obtuvieron puntuaciones más altas de envejecimiento exitoso. Estos hallazgos enfatizan la importancia de fomentar la participación productiva en la tercera edad y de mantener servicios de salud accesibles, pero que promuevan la independencia, para apoyar el envejecimiento saludable en contextos rurales.

Sin embargo, debido al diseño transversal de este estudio, no se pueden establecer relaciones causales. Además, también hemos observado que la proporción de participantes con respecto a los predictores puede limitar la estabilidad del modelo, por lo que los resultados deben interpretarse con cautela. Las investigaciones futuras deberían explorar enfoques longitudinales y cualitativos para comprender mejor los mecanismos subyacentes a estas asociaciones.

## Data Availability

El conjunto de datos completo que respalda los resultados de este estudio se publicó en el propio artículo. Se pueden solicitar datos adicionales al autor correspondiente previa solicitud razonable y con sujeción a consideraciones de confidencialidad.
